# Extracellular Hemoglobin: Modulation of Cellular Functions and Pathophysiological Effects

**DOI:** 10.3390/biom12111708

**Published:** 2022-11-17

**Authors:** Ivana T. Drvenica, Ana Z. Stančić, Irina S. Maslovarić, Drenka I. Trivanović, Vesna Lj. Ilić

**Affiliations:** 1 Group for Immunology, Institute for Medical Research, National Institute of Republic of Serbia, University of Belgrade, 11129 Belgrade, Serbia; 2 Group for Hematology and Stem Cells, Institute for Medical Research, National Institute of Republic of Serbia, University of Belgrade, 11129 Belgrade, Serbia

**Keywords:** extracellular hemoglobin, oxygen carriers, cell culture additives, hemolysis, differentiation

## Abstract

Hemoglobin is essential for maintaining cellular bioenergetic homeostasis through its ability to bind and transport oxygen to the tissues. Besides its ability to transport oxygen, hemoglobin within erythrocytes plays an important role in cellular signaling and modulation of the inflammatory response either directly by binding gas molecules (NO, CO, and CO_2_) or indirectly by acting as their source. Once hemoglobin reaches the extracellular environment, it acquires several secondary functions affecting surrounding cells and tissues. By modulating the cell functions, this macromolecule becomes involved in the etiology and pathophysiology of various diseases. The up-to-date results disclose the impact of extracellular hemoglobin on (i) redox status, (ii) inflammatory state of cells, (iii) proliferation and chemotaxis, (iv) mitochondrial dynamic, (v) chemoresistance and (vi) differentiation. This review pays special attention to applied biomedical research and the use of non-vertebrate and vertebrate extracellular hemoglobin as a promising candidate for hemoglobin-based oxygen carriers, as well as cell culture medium additive. Although recent experimental settings have some limitations, they provide additional insight into the modulatory activity of extracellular hemoglobin in various cellular microenvironments, such as stem or tumor cells niches.

## 1. Introduction

Hemoglobin, a highly conserved protein, due to its ability to reversibly bind oxygen, is involved in the processes that underlie the aerobic life on planet Earth. The primary role of this protein is reflected in the maintenance of cellular homeostasis, by supporting its energy requirements. However, due to nearly 200 years of research into hemoglobin, it is now known that this protein also plays important roles in cellular signaling and modulation of the inflammatory response. Hemoglobin performs these functions directly, by binding gas molecules (NO, CO and CO_2_), or indirectly, by acting as their source. In vertebrates, hemoglobin performs most of its functions while inside the erythrocytes [1,2,3]. However, once hemoglobin reaches the extracellular environment, in conditions such as trauma, inflammation or infection, hemoglobin exerts other potentially harmful effects on cells and tissues and may be involved in the etiology and pathophysiology of various diseases [4,5,6]. This dichotomy in function makes hemoglobin the so-called “two-edged sword”, a friend in physiological conditions, and an enemy or potential danger in stress conditions [1,6,7,8]. Furthermore, through applied biomedical research which implies application of exogenous hemoglobin originating from different sources, novel insights into the modulatory capacities of this macromolecule are obtained. Since the term “hemoglobin” represent a diverse protein superfamily based on the property to reversibly bind oxygen and to have a conserved heme-binding domain called the “globin fold” [9], this review covers data regarding both invertebrate and vertebrate extracellular hemoglobin influence on cells and tissue functional properties.

## 2. Extracellular Hemoglobin in Mammals

### 2.1. Endogeous Extracellular Hemoglobin Removal and Degradation

Under physiological conditions, about 80–90% of erythrocytes are destroyed without releasing hemoglobin into plasma, in a process termed extravascular hemolysis. The remaining erythrocytes are removed under physiological conditions by a process designated as intravascular hemolysis—hemolysis within blood vessels [8,10]. In intravascular hemolysis, hemoglobin is released directly into the circulation, where this molecule and its degradation products can cause cell and tissue damage [11,12] if they exceed the capacity of the mechanisms involved in their removal. Free hemoglobin in plasma is rapidly oxidized to methemoglobin, which readily and non-enzymatically dissociates into heme and αβ dimers. At low plasma hemoglobin release, haptoglobin (Hp) irreversibly binds all αβ-globin dimers present [13,14]. Hp binding sites are located on α-globin chains [15]. Hp is synthesized in parenchymal cells of the liver, and the half-life of this glycoprotein in the circulation is 3.5–5 days. However, if it binds αβ-globin dimers, the half-life of such a complex becomes only 9 to 30 min. These complexes are rapidly removed from the circulation by phagocytosis by monocytes and tissue macrophages after binding to their CD163 receptor [16]. Since there is no Hp recycling, once the reserves from circulation are depleted it takes 5–7 days for the pool to be recovered because a reduced Hp concentration does not lead to an increase in the synthesis of this molecule.

In addition to Hp, both hemopexin and albumin act as mechanisms that limit the effects of extracellular hemoglobin [3,14]. These two molecules can bind free heme, maintain it in soluble form and thus prevent it from exhibiting its oxidative and proinflammatory effects [17]. Hemopexin is a plasma heme-binding glycoprotein with an affinity for heme higher than all known heme-binding proteins. Moreover, hemopexin mediates the intake of heme in hepatocytes in which this prosthetic group is removed. Hemopexin is synthesized in the liver and in healthy individuals has a half-life of an average of seven days, while in the complex with heme, its half-life is reduced to 7–8 h [18]. The heme-hemopexin complex enters hepatocytes through receptor-mediated endocytosis via a lipoprotein receptor-related protein-1 receptor (LRP), also known as CD91. After the endocytosis, the heme-hemopexin complex dissociates, and the hemopexin is released and returned to the plasma as an intact protein. Transport of heme in the cytoplasm occurs by an internal heme-binding membrane protein, and iron is rapidly removed by the action of heme oxygenase [19,20].

Heme can also bind to albumin in the circulation and form methemalbumin. When added to human serum, heme is initially primarily bound to albumin, probably due to its high concentration relative to hemopexin. Removing methemalbumin from circulation is a kinetically complex process [17]. Previous research shows that plasma heme can also bind α1-microglobulin (A1M), a 26 kDa glycoprotein synthesized in the liver and secreted into the blood. This protein has reductase activity, prevents intracellular oxidation, and reduces the expression of heme-induced heme oxygenase-1 (HO-1) and reactive oxygen species (ROS) production due to the presence of hemoglobin in the extracellular environment [21,22,23]. The newest data suggest the use of apohemoglobin or apohemoglobin-Hp as a novel therapeutic strategy in scavenging and clearing excess heme through the monocyte/macrophage CD163 surface receptor [24]. Subramanian et al. [25] identified a “shortcut” for detoxification of extracellular hemoglobin within plasma, which functions Hp-independently via the capture and quench mechanism and involves the CD163 monocyte/macrophage receptor. Following hemoglobin recruitment, membrane CD163 (mCD163) directly suppresses the pseudoperoxidase activity of hemoglobin in situ on the monocyte membrane. Hemoglobin induces the release of mCD163 into plasma, and the resulting soluble CD163 (sCD163) further captures and quenches residual redox-reactive hemoglobin. These authors showed that sCD163 and immunoglobulin G (IgG) interact with extracellular hemoglobin in plasma. The resulting sCD163-Hb-IgG complex then triggers an autocrine loop of endocytosis via Fcγ receptors on monocytes and consequent recycling of internalized sCD163 via endosomes to establish mCD163 homeostasis, while internalized hemoglobin is catabolized by HO-1. Additionally, this complex induces paracrine transactivation of vascular endothelial cells, stimulates HO-1 expression in them, and secretes cytokines that will trigger a systemic defense response directed toward extracellular hemoglobin [25].

### 2.2. Endogenous Extracellular Hemoglobin Mode of Action

**Figure 1:**
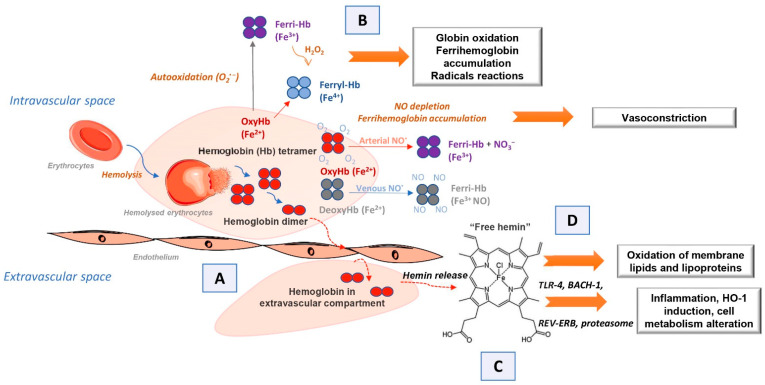
Scheme illustrating the mechanisms of action of endogenous extracellular hemoglobin: (**A**) extravascular translocation of hemoglobin, (**B**) prooxidative reactivity of hemoglobin in plasma or within tissues after extravasation, (**C**) release of hemin from Hb-Fe^3+^ as the main product of oxidative reactions and (**D**) hemin induced changes in cell activation, gene expression, and metabolism.

If extracellular hemoglobin exceeds the homeostatic mechanisms for its removal during intravascular hemolysis, it can affect surrounding cells and tissues. Various clinical aspects associated with circulating extracellular hemoglobin excess have been attributed to hemoglobin molecule-specific structural and biochemical characteristics through four proposed interacting mechanisms [1,11]. The first of these is the extravascular translocation of hemoglobin (Figure 1A) [11]. After hemolysis, hemoglobin exists in the dynamic equilibrium of tetramers and αβ heterodimers, with a predominant dimer state at low plasma hemoglobin concentrations. Heterodimers are relatively small and capable of translocation and access to vulnerable anatomical sites (for example, glomeruli in the kidneys or vascular wall). Tissue exposure to hemoglobin most often accompanies cases of excessive hemoglobinuria after massive intravascular hemolysis, but hemoglobin can also translocate through endothelial barriers and thus enter the subendothelial and perivascular spaces and lymph [26,27]. The hemoglobin dimer/oligomer is considered a damage/danger-associated molecular pattern (DAMP), while heme is independently recognized as an “alarmin” [28,29,30].

Another mechanism by which extracellular hemoglobin exerts its effects is the prooxidative reactivity of hemoglobin in plasma or within tissues after extravasation (Figure 1B) [11]. The reactions of hemoglobin with NO and physiological oxidants (hydrogen peroxide and lipid peroxides) are best studied. NO consumption and consequent oxidation of hemoglobin occur through two reactions: (1) NO dioxygenation of oxyhemoglobin in which nitrates (NO_3_^−^) and ferrihemoglobin (Hb-Fe^3+^) are generated (Equations (1) and (2)) through nitrosylation of iron deoxyhemoglobin which occurs in direct binding of NO to iron of ferrohemoglobin (Hb-Fe^2+^) [11,31,32]. The binding of NO by cell-free hemoglobin leads to depletion of this critical vasodilator produced in the vascular endothelium; it is reported that this reaction is the fundamental cause of hypertension [32], since even the recombinant hemoglobin, with diverse chemistries [33], exerts a similar rate of reaction (Equation (1)). The reaction of extracellular hemoglobin and NO also results in the generation of Hb-Fe^3+^ within the tissue parenchyma. The accumulation of Hb-Fe^3+^ can stimulate the release and/or transfer of hemin to other proteins and lipids, thus manifesting the secondary toxicity of free hemin. Although hemoglobin and peroxide reactions have been the subject of numerous studies for over 40 years, the pathophysiology underlying these reactions is still not completely clarified [2,11,19,34].

HbFe^2+^ + NO → NO_3_^−^ + HbFe^3+^ (1)

It has been shown that in vitro, the reaction of hemoglobin and peroxide leads to the formation of Hb-Fe^3+^ and also to chemical species in which iron is in the ferryl form (Hb-Fe^4+^) and its radical forms (Equations (2) and (3)) [4,32,35,36].

HbFe^2+^ + O_2_ → HbFe^3+^ + O_2_^−^ (2)

HbFe^3+^ + H_2_O_2_ → ^•^HbFe^4+^ =O + H_2_O (3)

The outcome of such reactions is hemoglobin degradation, hemin loss, cross-linking, or precipitation of globin chains, which lead to tissue damage. It is still unknown how much Hb-Fe^4+^ and radicals are formed in vivo during hemolysis and to what extent they contribute to the development of the disease. The only oxidized type of hemoglobin that can be consistently quantified in vivo is Hb-Fe^3+^. In other words, it should be taken into account that the disparity observed in vitro and in vivo may be due to a shifted balance between oxidation and reduction reactions in vivo [34,37]. The third proposed mechanism by which extracellular hemoglobin exerts its effects is the release of hemin from Hb-Fe^3+^ as the main product of oxidative reactions (Figure 1C) [11]. The release of hemin allows the transfer of reactive porphyrin to the cell membrane or soluble plasma proteins and lipids and provides free hemin as a ligand for various signaling pathways. As hemin is a hydrophobic molecule, it is unlikely that more significant amounts of free hemin will be found in plasma; it binds rapidly to albumin or lipids and forms complexes. Depending on what it binds to, hemin can transform a molecule from a complex into a reactive product, such as oxidized low-density lipoprotein, further damaging the vasculature [8,38,39]. The fourth mechanism refers to the effects achieved by hemin (Figure 1D) [11]. Hemin can selectively bind to several receptors and transcription factors and lead to changes in the state of cell activation, gene expression, and metabolism. The best-characterized interaction is hemin binding to transcriptional repressor 1, which shows homology to BTB and CNC homology 1, Bach-1, which regulates the transcription of HO-1 and other antioxidant enzymes necessary for an adaptive response to increased intracellular hemin levels [40]. Hemin is also a ligand for the nuclear receptor of the hormone REV-ERB, which regulates circadian rhythm, glucose metabolism, and adipogenesis [41].

## 3. Exogenous Administration of Extracellular Hemoglobin

**Figure 2:**
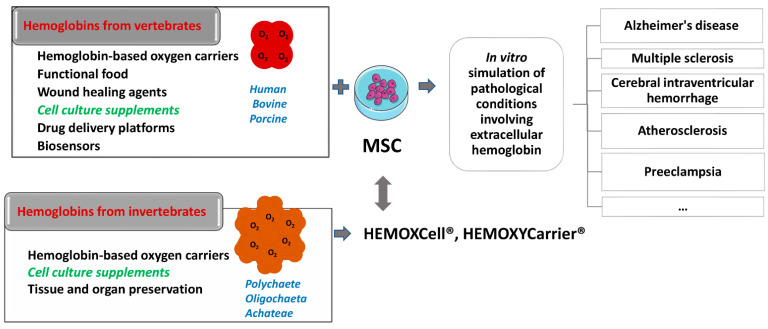
Schematic presentation of the potential use of vertebrates and invertebrates’ hemoglobin preparations in biomedicine and biotechnology (left side of the scheme), emphasizing already commercially available cell culture additives based on invertebrate hemoglobin (HEMOXCell^®^, HEMOXYCarriers^®^, HEMARINA SA, Morlaix, France). Up-to-date research on the impact of such hemoglobin preparation on mesenchymal stromal cell (MSC) cultures could significantly contribute to a better understanding of the onset and progression of pathological conditions involving extracellular hemoglobin as presented on the right side of the scheme.

The very first beginnings of hemoglobin application in biomedicine are related to its use as a blood substitute, i.e., more precisely as oxygen carriers (hemoglobin-based oxygen carriers, HBOCs) [32,42,43,44] (Figure 2). The first HBOC was hemoglobin isolated from lysed erythrocytes given to patients intravenously. However, it has been observed that soon after applying hemoglobin solution in this way, besides its short intravascular persistence, kidney damage, high blood pressure, and cardiovascular complications occur [45,46,47] due to the effects of extracellular hemoglobin. These features and mechanisms of the act of extracellular hemoglobin are well known today and explained in detail in several comprehensive reviews and in the previous sections. Stroma-free hemoglobin rapidly dissociates into dimers and monomers, activates liver and spleen macrophages, and has the ability to bind the potent vasodilator, NO, resulting in vasoconstriction [32]. Due to hemoglobin dimer precipitation in proximal tubules, nephrotoxic action occurs. Additionally, outside of erythrocytes, hemoglobin quickly oxidizes to methemoglobin, which cannot bind oxygen; instead, it releases heme, which participates in the creation of free radicals that exert harmful effects on surrounding cells [43,44,48,49]. After such reports on outcomes of extracellular hemoglobin administration, researchers have focused on obtaining recombinant hemoglobin by mutagenesis with desired features and reduced side effects. With the use of recombinant technology, a low oxygen affinity human hemoglobin variant was produced in *Escherichia coli* by Somatogen Inc. by introducing the substitution of amino acid Lys for Asn at the β108 position [32]. To further stabilize this hemoglobin in tetrameric form, a site-specific mutation was introduced by inserting glycine into the region where two α subunits almost touch each other, fusing them (rHb1.0) [50]. The second generation of recombinant hemoglobin (rHb2.0) included a change of heme pocket chemistry by introducing site-directed mutagenesis followed by polymerization [51], such as providing HbA mutants with lower NO reactivity. A comprehensive summary of the impressive work done by biochemists in this field of recombinant hemoglobin is disclosed in the review of Varnado et al. [52]. Progress in the development of recombinant hemoglobin enabled (i) adjustment of oxygen (O_2_) affinity over a 100-fold range, (ii) reduction of nitric oxide (NO) scavenging activity over 30-fold without compromising dioxygen binding, (iii) decrease of antioxidant activity, (iv) reduction in hemin loss rate, (v) regulation of subunit dissociation rate, and (vi) decrease of irreversible subunit denaturation [52]. Since the relatively high cost of large-quantity production of these proteins for emergency management of trauma patients is the main obstacle for their further progress, more cost-effective chemical modifications of the hemoglobin molecules have been launched [43,53,54,55,56,57,58,59,60,61,62,63]. The procedure includes hemoglobin cross-linking/polymerization with succinyldisalicylic acid [54], glycine [55], glutaraldehyde [56], *O*-raffinose [57,58] or modification of the surface area of hemoglobin molecules with polyethylene glycol (PEG) [59,60] or human serum albumin with bound platinum nanoparticles [61,62]. Another successful approach towards overcoming the side effects of a “naked hemoglobin molecule” administration involves the use of encapsulation as a method. Hemoglobin encapsulated in different biomaterials revealed longer circulatory lifetime, decreased hypertensive response and phagocytic uptake [43]. From the pioneering steps in this approach by Chang et al. [63], encapsulating material for hemoglobin varied from cellulose nitrate or PEG-polylactide to poly(ε-caprolactone)/poly(l-lactic acid) and poly(l-lysine), poly(lactic-coglycolicacid)/PEG copolymers and various lipid vesicles [43]. Encapsulation of hemoglobin within emulsion with hydrophobic nanodrops (where hydrophobicity features of the nanoemulsion structures influence the binding of the hydrophobic niche of hemoglobin and thus partially act as a “stunt” -2, 3-DPG moderator) is patented demonstrating very promising results in the model of hemorrhagic rats [64]. Recent studies on hemoglobin encapsulation in lipid vesicles demonstrated remarkable progress by means of larger-scale production without affecting the biophysical properties of hemoglobin molecules [65] or introduction to clinical trials [66].

Both allogeneic and xenogeneic hemoglobins were used as a source of extracellular hemoglobin. Among xenogeneic hemoglobins, the most studied are bovine and porcine hemoglobin, which show a high degree of homology with human hemoglobin (84.4% and 85%, respectively). Apart from the use as blood substitute, porcine hemoglobin was used as a wound healing agent in the form of spray if applied at regular intervals of 2–4 weeks. Namely, this product has been shown to reduce pain, wound size, and the necrotic tissue layer that impairs wound healing [67,68]. Moreover, there are reports on bovine hemoglobin used as a drug platform itself, i.e., as a pH-sensitive nano-vehicles for potential cancer detection and therapy [69] and as an effective glucose biosensor in vitro [70]. One part of the research relies on examining rodent hemoglobin as well, aiming to simulate extracellular hemoglobin presence in vivo [71]. Furthermore, hemoglobin from invertebrates has also become a significant subject of research. Thus, potential of extracellular hemoglobin as an additive for organ and tissue preservation [72,73] and as a growth stimulator of mesenchymal stromal cells (MSC), which maintain ‘‘stemness” in vitro [74], have been described. Therefore, one should be aware that under the same term “hemoglobin”, the studies on extracellular hemoglobin functions imply both vertebrate and evolutionary distant invertebrate hemoglobin.

Among invertebrate hemoglobins, the most investigated are extracellular hemoglobin of annelids (also named erythrocruorins and chlorocruorins after the nature of the porphyrin group) [75,76], present in three classes of annelids: *Polychaete*, *Oligochaeta*, and *Achateae*. Annelid hemoglobins are huge biopolymers with a high molecular weight of 3000–4000 kDa [76,77]. The beginning of the application of EHb in biotechnology is related to the group led by Andre Tulmond and Franck Zal. This research group started an examination on hemoglobin from the marine polychaete *Arenicola marina* (HbAm) in 1993 and described the structure of several extracellular hemoglobins in detail [78]. It has been discovered that the hemoglobin of *Arenicola marina* (HbAm) had all the sought traits of the universal oxygen carrier: it is naturally extracellular and polymerized, and its molecular mass is 50 times greater than the mass of human hemoglobin. It had properties of O_2_-binding and release like human hemoglobin within erythrocytes. Each HbAm molecule can bind 156 oxygen molecules (human Hb can bind four) and has natural antioxidant properties. Additionally, HbAm has functional properties independent of secondary molecules such as 2,3-DPG in humans. The HbAm molecule displayed activity without any additional chemical modifications. It appears to be stable at temperatures ranging from 4 to >30 °C and does not show any vasoconstriction effects upon application [76,77]. HbAm dissociates in human plasma from a hexagonal bilayer into globin dodecamers (although the dodecamer still appears to function as desired without extravasating) [79,80]. It is important to note erythrocruorin of *Lumbricus terrestris* (LtEc), which has been shown to be extremely stable, resistant to oxidation, and may interact with NO differently than mammalian hemoglobin molecules [80].

## 4. Evidence on Capacity of Mammalian Endogenous and Exogenous Extracellular Hemoglobin to Regulate Cellular Functions and Molecular Signaling in Health and Disease

Extracellular hemoglobin can modulate healthy tissue cells and tumor cells, where hemoglobin removal is recognized as an essential strategy for the prevention of hemoglobin secondary roles display. As stated in Section 2, hemoglobin is metabolized by tissue macrophages, and the primary sources are senescent (extravascular hemolysis) or damaged erythrocytes (intravascular hemolysis). Complex Hb-Hp binds to the scavenger receptor cysteine-rich domain protein CD163 undergoing endocytosis and degradation by lysosomes [36,81]. Despite this, the aging process and renewal of the erythrocyte pool are followed by the release of small amounts of hemoglobin into circulation, but also under certain pathophysiological conditions, blood transfusion, or administration of hemoglobin-based carriers of oxygen [36,42].

**Figure 3:**
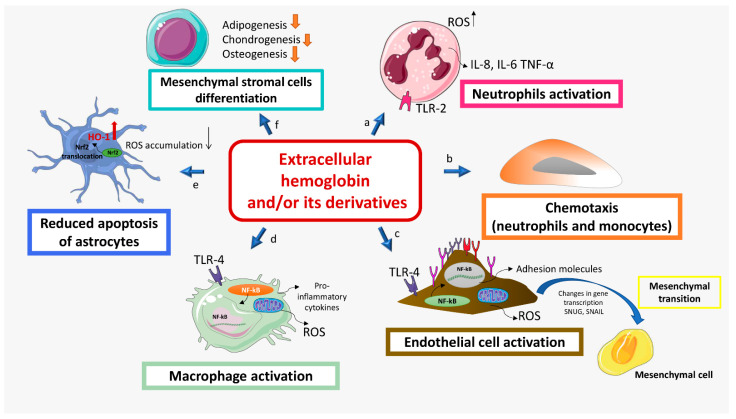
Targets of extracellular hemoglobin and its derivatives relevant for pathophysiology of several diseases (see Section 4). Extracellular hemoglobin and/or its derivatives induce (**a**) neutrophil activation characterized by elevated ROS production, increased production of pro-inflammatory cytokines, (**b**) neutrophil and monocytes chemotaxis, (**c**) endothelial cell activation characterized by NF-κB activation, elevated ROS production, and increased expression of adhesion molecules and pro-inflammatory cytokines. (**d**) Macrophages elevated ROS production and pro-inflammatory cytokine production, (**e**) astrocytes reduced ROS accumulation and apoptosis rate characterized by upregulation and nuclear translocation of Nrf2 and HO-1 upregulation, (**f**) MSC reduced osteogenesis, chondrogenesis and adipogenesis. (NF-κB, nuclear factor kappa B; ROS, reactive oxygen species; TLR2, toll-like receptor 2; TLR4, toll-like receptor 4, Nrf2, erythroid 2 related factor; HO-1 hem oxygenase-1; MSC-mesenchymal stromal cells).

There is growing evidence of the role of extracellular hemoglobin and its derivatives in the etiology and pathophysiology of numerous diseases. Although all these diseases have their unique symptoms, one in common is hemoglobinemia excess of extracellular hemoglobin in the blood plasma. The list includes paroxysmal nocturnal hemoglobinuria [82], sickle cell anemia [4,83,84], thalassemia, spherocytosis [85], Alzheimer’s disease [86,87,88,89], multiple sclerosis [35,90,91], cerebral intraventricular hemorrhage [92], preeclampsia [21,93,94], acute respiratory distress syndrome [95], thrombotic microangiopathy, acute kidney injury, chronic kidney disease, and atypical hemolytic uremic syndrome, among others [85]. In addition, it is worth acknowledging that several reports recorded increased bilirubin in COVID-19 patients, indicating the involvement of extracellular hemoglobin in disease severity via increased formation of carboxyhemoglobin [96]. From the existing findings, it can be summarized that extracellular hemoglobin can impact: (i) redox status, (ii) inflammatory state of cells (iii) proliferation and chemotaxis, (iv) mitochondrial dynamic, (v) chemoresistance and (vi) differentiation. These features of extracellular hemoglobin will be described via several of the most specific pathological states (Figure 3).

In diseases characterized by hemoglobinemia, the incidence of thrombosis is increased [82]. Hemolysis stimulates coagulation through several mechanisms, including phosphatidylserine exposure to the erythrocyte surface, accumulation, and depletion of NO by free hemoglobin, endothelial dysfunction, and increased tissue factor (TF) expression. Intravascular coagulation results in fibrin deposition, which causes hemolysis, creating mechanical damage to erythrocytes. Roth showed that while hemoglobin stimulates LPS-induced TF production in endothelial cells, it does not induce TF expression in these cells [97]. However, heme can induce TF production, as was shown using endothelial cells as cell models [83]. It appears that hemoglobin can impact redox homeostasis of cells. Namely, the expression of TF was upregulated in macrophages by hemoglobin, and hemoglobin desensitized TF to the effects of antioxidants like glutathione or serum [10]. It has been shown that leukocytes in whole blood incubated with extracellular hemoglobin for 4 h at 37 °C, 5% CO_2_, and 95% humidity release proinflammatory IL-6, IL-8, and TNF-α [98]. In the same study, the authors reported inhibition of cytokine release by hydrocortisone, suggesting that the inclusion of anti-inflammatory compounds in hemoglobin solutions may prevent undesirable effects caused by inflammation after its infusion [98].

Several studies have shown that hemoglobin can interact with microbial ligands: pathogen-associated molecular patterns (PAMP), DAMP, and TLRs [12,19,99]. It has been shown that there are lipopolysaccharide (LPS) binding sites on α- and β-globin chains and that binding to this molecule leads to a change in the structure of hemoglobin that allows peroxidase activity [10]. Methemoglobin, alone or combined with lipoteichoic acid (LTA), can be recognized by TLR-2 on the neutrophil cell membrane [100] (Figure 3a). Such an interaction stimulates neutrophil function, initiating signal transduction of nuclear factor kappa B (NF-κB), which culminates in the synthesis of cytokines and other pro-inflammatory agents [101]. Endothelial cells can also detect extracellular hemoglobin via the TLR-4 receptor and signaling pathway. Free hemin can trigger the TLR-4 signaling pathway [18,102] and upregulate the transcription factors SNAI1 and SLUG responsible for endothelial to mesenchymal transition, as demonstrated on pulmonary artery endothelial cells [66] (Figure 3c).

Reported results from the studies that mainly recruited rodent models show that understanding different extracellular hemoglobin-metabolite roles is crucial for development of neuroprotective strategies based on free hemoglobin-metabolite scavengers. Data reported up to now described the association of endogenous hemoglobin increase and brain damage, while details on hemoglobin-metabolites responsible for the brain damage are still conflicting. Namely, it has been observed that oxyhemoglobin in low concentrations reduced oxidative stress and caspase activation and thus protects cortical rat astroglial cell cultures exposed to hydrogen peroxide [103]. The authors suggested that oxyhemoglobin was able to induce protein kinase A and C signal transduction pathways along with reduced NF-κB pathway activation. In vitro exposure of an immature primary rat mixed glial cell culture to oxidized hemoglobin led to a similar damaging response as exposure to hemorrhagic cerebrospinal fluid, where the rate of ROS production positively correlates with that of pro-inflammatory cytokines. Moreover, oxidized hemoglobin caused structural disintegration in the mixed glia cells, while Hp only partially abolished these damaging effects [104]. Besides the impact of hemoglobin on the inflammatory and redox status of cells, recent studies revealed that hemoglobin can influence mitochondria as well. In a recent study, utilizing oligodendrocyte progenitor cells (OPCs) showed that exogenous extracellular hemoglobin induced mitochondrial dysfunction in OPCs and that antioxidant compound could reduce these effects; according to this study, hemoglobin induced oxidative stress and impaired mitochondrial function in OPCs isolated from Sprague Dawley rat pups [105]. Since globin chains are shown to be expressed in cortical and hippocampal astrocytes in the corpus striatum, corpus callosum, substantia nigra, and medulla oblongata [106] and other regions of the brain [90,107], many researchers focused their attention on the role of hemoglobin in the pathophysiology of Alzheimer’s disease, and many of these studies were conducted using rodent cell models. It was shown that HO-1 protects cortical astrocytes from oxidative death caused by exposure to micromolar concentrations of extracellular hemoglobin [108]. By hypothesizing that hemin, as the product of hemoglobin degradation, is responsible for hemoglobin toxicity via the induction of reactive oxygen species, other authors showed that hemoglobin-pretreated astrocytes in culture were resistant to hemin-induced toxicity [109]. HO-1 was once again confirmed as a molecular mechanism involved in this process, along with nuclear transcription factor-erythroid 2 related factor (Nrf2). The results showed that hemoglobin induced upregulation and nuclear translocation of Nrf2 in astrocytes and resulted in HO-1 upregulation, which contributed to reduced ROS accumulation and apoptosis rate (Figure 3e). Some studies showed that the concentration of globin chains in patients with Alzheimer’s disease is lower in neurons compared to healthy people [86,88]. However, some reports indicate the increase in hemoglobin expression in brain homogenates in patients with Alzheimer’s disease [110]. It is assumed that this discrepancy in the literature data reflects different roles of extracellular and intracellular hemoglobin in the nervous system. While intracellular hemoglobin protects neurons from hypoxia, extracellular hemoglobin and its degradation products can damage the blood-brain barrier allowing blood to enter the brain. Chronic hemolysis leads to saturation of the extracellular hemoglobin detoxification system, thus accumulating free hemoglobin around cerebral micro blood vessels [89].

Numerous studies explore the role of extracellular hemoglobin in multiple sclerosis etiology. In patients with multiple sclerosis, iron has been shown to accumulate in dilated veins proximal to demyelinated plaques in gray and white matter [111]. Several scenarios can lead to this: abnormalities in the functioning of the blood-brain barrier, inefficient iron removal or chronic subclinical cerebral microbleeds [35,90,91]. Using an oligodendroglial cell line, Bamm et al. showed that hemoglobin leads to oxidative damage to myelin proteins and lipids and suggested a mechanism by which free extracellular hemoglobin can invade the central neural system parenchyma due to intravascular hemolysis or erythrocyte extravasation followed by their lysis [35,91]. Furthermore, hemoglobin, as a highly reactive molecule, can lead to local oxidative stress, inflammation, and tissue damage.

The role of extracellular hemoglobin has also been investigated in the etiology and progression of atherosclerosis, a well-known multifactorial inflammatory disease in which inflammatory and immune responses are critical pathophysiological factors. Bleeding within atherosclerotic plaque is a frequent event that leads to the release of free hemoglobin and the activation of an Hp removal system. Buttari et al. showed that in the presence of a mixture consisting of 18% oxyhemoglobin, 3% methemoglobin, and 79% hemichrome, the expansion of hemoglobin-specific T lymphocytes, which secrete IFN-γ, occurs in patients with advanced carotid atherosclerosis [112]. The same mixture promoted the maturation of LPS-stimulated dendritic cells, which was confirmed by the detection of the maturation marker CD83 and costimulatory molecules CD80, CD40, human leukocyte antigen (DR isotype, HLA-DR), and CD86 molecule, on dendritic cells. The same authors showed that hemoglobin acts as a chemoattractant for monocytes and dendritic cells, directing them towards the vascular wall [113] (Figure 3b). To further investigate the mechanism by which extracellular hemoglobin performs this role, Posta et al. showed that atherosclerotic lesions accumulate peptides derived from hemoglobin and ferryl hemoglobin which induce intercellular gap formation, decrease junctional resistance in the endothelium, and enhance monocyte adhesion to endothelial cells [114].

Centlow and colleagues have shown that there is stimulation of fetal hemoglobin (HbF) gene expression and accumulation of extracellular fetal hemoglobin in the vascular lumen of the placenta in women with preeclampsia [115]. In addition, Olson et al. have shown that women with preeclampsia have increased levels of plasma HbF and extracellular hemoglobin A [116]. It is assumed that local placental hypoxia is induced in this condition, which further causes the expression of placental HbF genes and proteins. Extracellular HbF leads to ROS formation and oxidative damage and to leakage through the fetomaternal barrier. This leakage results in an increased concentration of HbF in the mother’s plasma and further induction of ROS formation, followed by endothelial dysfunction, hypertension, and proteinuria, which are the main symptoms of preeclampsia.

An additional set of findings revealed the role of hemoglobin in modulation of cellular response to chemotherapeutics. Namely, it was reported that due to intratumoral hemorrhage, hemoglobin could, as an endogenous danger signal, promote tumor cell proliferation and growth of breast cancer and melanoma in a syngeneic mouse model. Namely, erythrocyte and hemoglobin activated the ROS–NF-kB pathway in both tumor cells and macrophages. Moreover, along with elevated ABCB1 gene expression, erythrocytes and extracellular hemoglobin induced chemoresistance in tumor cells and accumulation of anti-inflammatory macrophages [117] (Figure 3d). The aforementioned data indicate a very complex role of endogenous accumulation of extracellular hemoglobin as well as exogenous hemoglobin treatments. Namely, recently Lucas et al. [118] showed that co-administration of polymerized hemoglobin with cisplatin in an animal model attenuated tumor growth without alleviating hypoxia [118], where most probably the exogenous ROS production by oxidized polymerized hemoglobin is the mechanism of chemo-sensitization [118].

Being a potential modulator of both epithelial and hematopoietic systems, the inevitable question is whether and how extracellular hemoglobin and hemoglobin-metabolites impact cells of mesenchymal lineage. In the next paragraphs we discuss current models of the involvement of extracellular hemoglobin in different pathologies and how it can potentially contribute to the functioning of tissues of mesodermal origin, including modulation of mesenchymal stromal cell (MSC) features. The influence of extracellular hemoglobin on MSCs has gained the attention of the scientific community in the last few decades due to their regenerative potential, based on self-renewal abilities and migratory and differentiation potential [119,120,121]. MSCs participate in maintaining homeostasis in the body thanks to the interaction with various components of the microenvironment in which they are located, including cells of the immune system [122,123,124,125]. In addition, MSCs are known to respond to inflammatory signals of the microenvironment, as well as to act as immunomodulators [126,127,128].

It appears that free hemoglobin can attenuate rat cartilage growth in a dose-dependent manner, which might explain cartilage retardation under chronic hemolytic conditions [129]. Moreover, it has been found that increased bilirubin concentration (product of free hemoglobin) can decrease the thickness of femoral cartilage in patients suffering from sickle cell disease [130]. Moreover, a study on synovial tissues derived from osteoarthritis, rheumatoid arthritis and meniscus injury patients revealed that free hemoglobin produced by intra-articular hemorrhage stimulates the secretion of uPA, matrix metalloproteinases (MMP-2 and MMP-9) by synovial cells as well their fibrinolytic activity and gelatinolytic activity; the results were also confirmed in a rabbit model in vivo [131]. From these findings we can conclude that extracellular hemoglobin might impair cartilage growth due to regulation of chondrogenic differentiation of MSCs.

In our investigation we tested effects of bovine hemoglobin (BHb) and porcine hemoglobin (PHb) in the culture of primary MSCs isolated from human peripheral blood (PB-MSCs) and mouse mesenchymal cell lines. BHb and PHb have strong homology to human adult Hb, and therefore their usage in human culture systems is meaningful [71,132]. Both BHb and PHb can be obtained from slaughterhouse blood erythrocytes [133]. Our study showed that bovine Hb (BHb) can impair proliferation of MSCs isolated from human peripheral blood (PB-MSCs), and this effect appears to be dose-dependent [71]. On the other side, there was no significant effect achieved by PHb on PB-MSC viability [134]. Even at the lowest concentration applied (0.1 µM), which did not affect proliferation, BHb and PHb reduced clonogenic growth, osteogenic and chondrogenic differentiation in vitro, along with regulated expression of genes involved in differentiation programs (*Runx2*, *Alpl* and *BGLAP*, *Sox9*, *Col2a1*, *Col1a1*) [71,134] (Figure 3f). Interestingly, it appears that MSCs can be affected by iron overload due to hemoglobinopathies. Addition of iron (at concentrations estimated in sera of iron overloaded patients) to BM-MSCs induced their entry into S-phase and proliferation but reduced osteogenic differentiation. In line with this is that bone alterations, such as osteoporosis and fractures, have been documented in hematologic iron overload diseases and in animal models [135]. Together, these results are in accordance with previously discussed participation of Hb in regulation of bone and cartilage tissue health. However, this evidence suggests a potential loss of stem cell fitness of MSCs, where in the future it will be necessary to reveal whether these changes are transient and whether their stem cell features can be recovered.

Moreover, BHb and PHb increased PB-MSC motility in a dose-dependent manner. On the other side, an impact on adipogenesis was observed at the transcriptional level only, where reduced *PPARG* and *AdipoQ* mRNA in PB-MSC were demonstrated. Except for clonal growth and motility, similar effects were confirmed in corresponding mouse cell models for osteogenesis (M3T3-E1), chondrogenesis (ATDC5) and adipogenesis (3T3-L1) [71]. According to this, impaired murine BM-MSC functions were reported in mouse sickle cell disease [136] and β-Thalassemia [137] models, where BM-MSCs displayed impaired hematopoiesis support, activated inflammatory TLR-4 signaling, increased ROS production, reduced differentiation, and a senescent phenotype. These results indicate that both BHb and PHb can represent a reliable extracellular form of hemoglobin where the effect on main cellular functions appears to be consistent in human and mouse cell systems.

Similarly, our group compared the effects of PHb and BHb in the C2C12 cell line as a model of satellite cells and myogenic differentiation, and although we found dose-dependent trends in both hemoglobin groups when investigating the proliferation rate, BHb showed the stronger effect [138]. However, both PHb and BHb decreased the expression levels of myogenin and muscle-specific creatine kinase; moreover, a certain impact was observed when the area and length of C2C12 myotubes were analyzed. In addition, the results showed increased expression of Hif1-α, without change of *Hmox1* mRNA expression [138]. Thus, it appears that Hb can modify myogenesis as well, and further investigation should be conducted to elucidate the importance of Hb for muscle-related diseases or injuries. Taken together, Hb strongly regulates differentiation in MSCs and other progenitor cells, and follow-up studies are required to reveal whether Hb simultaneously can damage stem cell capacity (e.g., self-renewal) and how its activity is coordinated in a timeframe. Importantly, observed effects are in line with the abovementioned data on the impact of endogenous hemoglobin in homeostasis and diseases. The results of our studies on the effects of PHb and BHb [71,134,138] support the assumptions that extracellular hemoglobin can participate in modulation and redirection of MSC differentiation capacity (along with other inflammatory mediators) found in local inflammatory states (e.g., periodontal diseases) or some systemic inflammatory conditions accompanied by extravascular hemolysis. Therefore, we anticipate that by using extracellular exogenous BHb and PHb and appropriate mesenchymal cells, it should be possible to design a reliable in vitro model relevant for obtaining fundamental knowledge of extracellular hemoglobin-mediated cellular response and disease modelling. Future directions of our in vitro trials will include variation of hemoglobin concentration and several encapsulation techniques to achieve a more effective delivery of hemoglobin to the cell culture or more reliable 3D cultures, such as multicellular organoids.

## 5. Evidence of Capacity of Invertebrate Hemoglobin to Regulate Cellular Functions and Molecular Signaling

As introduced in Section 3, starting from the end of the 20th century, natural acellular hemoglobin, which provides oxygen transport and delivery within many terrestrial and marine invertebrates, is recognized as a model of hemoglobin-based oxygen carriers (HBOC) due to demonstrated distinctive biophysical characteristics [80,139,140,141]. *Lumbricus terrestris* erythrocruorin (LtEc) is a naturally occurring high molecular weight protein assembly (3.6 MDa) that is extremely stable, resistant to oxidation, and transports oxygen similarly to human whole blood [142]. Elmer and co-workers demonstrated that LtEc may be easily purified and safely transfused into hamsters using a hypervolemic infusion model in small amounts (0.5–1.5 g/dL final concentration in blood) without any noticeable side effects. LtEc did not elicit hypertension or vasoconstriction when infused into hamsters and effectively bound and released O_2_, with a high Hill coefficient and O_2_ affinity, similar to human whole blood [143]. Furthermore, LtEc maintained blood pressure without inducing vasoconstriction, while increasing microvascular perfusion and functional capillary density relative to Dex70 and human serum albumin in a model of hamsters with severe anemia [144]. Previous studies have also demonstrated that no immune and toxic response was observed even after repeated injections of LtEc to mice and a rat model and that it serves as a perfusion agent, significantly improving oxygen delivery to guinea pig hearts. [139].

With regard to commercialization of invertebrate hemoglobins, a great leap was made by Frank Zal and Morgan Rousselot, who founded a biotechnology company called HEMARINA in March 2007 to continue developing HbAm as a third-generation blood substitute [76,77,79]. One of the HEMARINA products is HEMO2life^®^ (Morlaix, France), intended for tissue and organ preservation. The main problem in organ transplantation is how to prolong organ ex vivo viability to enable their transport. The lifetime of the transplant is limited by harmful side products, primarily free radicals, which occur due to disruption of circulation (ischemia). At physiological temperature (37 °C), these free radicals lead to rapid necrosis and tissue deterioration and are transmitted to the recipient by perfusion of the transplanted organ. Because HbAm is functional in the natural environment at low temperatures, HEMO2life^®^ allows organ oxygenation when added to several clinical organ storage media. The effectiveness of HEMO2life^®^ has been shown for the lungs, liver, pancreas, kidneys, and heart in a large pig model. In addition, HEMO2life^®^ has intrinsic superoxide dismutase activity that protects cells from oxidative stress, further increasing the storage efficiency of transplant organs [76,77,145]. Additionally, no immunological, allergic or prothrombotic effects were observed with HEMO2life^®^ application. The newest data on the testing of HEMO2life^®^ showed that when added to the preservation solution it protected fatty livers during static cold storage (SCS) by decreasing reperfusion injury and improving graft function [146]. Transaminases, glutamate dehydrogenase and lactate levels at the end of reperfusion were significantly lower in the group of animals preserved by HEMO2life^®^. It also contributed to less inflammation in this group and less tissue damage compared to the control one. Although performances of a graft oxygenated during preservation by HEMO2life^®^ added to SCS (hemoglobin derived from *Arenicola marina* (M101) were not as good as that preserved by a hypothermic oxygenated perfusion machine), M101 effectively oxygenated liver grafts during preservation and prevented post-transplant injury [147]. Lupon et al. even hypothesized that HEMO2life^®^ can be characterized as a “molecular respirator” which could improve COVID-19 patient survival, avoid tracheal intubation and shorten oxygen supplementation [148]. This would open the possibility of treating a larger number of patients in the event of a lack of respirators, or perhaps instead of a respirator as an invasive device.

Another product made of invertebrate hemoglobin is HEMOXYCarrier^®^ (Figure 2) which is developed as HBOC. Intravenous administration of this product enables fast, efficient, and easy restoration of oxygenation capacity after massive blood losses in animal models, without side effects. This molecule is stable at room temperature and can be lyophilized. Preclinical studies of this product in rodents have shown that this molecule does not trigger immune responses, has no vasoconstriction effect, and does not affect arterial blood pressure [76,79]. HEMOXYCarrier^®^ decreases *Porphyromonas gingivalis*-induced inflammation in human oral epithelial cells [149]. Added to cell culture, this oxygen carrier significantly down-regulated pro-inflammatory cytokines (interleukin 1β (IL-1) and IL-8) and chemokine ligands (chemokines RANTES (regulated upon activation, normal T cell expressed and presumably secreted)) and IP-10 (interferon gamma-induced protein 10). Simultaneously, it up-regulated pro-healing mediators (PDGF-BB (platelet derived growth factor BB), TGF-β1 (transforming growth factor beta 1), IL-10, IL-2, IL-4, IL-11 and IL-15, and extracellular and immune modulators (TIMP-2, TIMP metallopeptidase inhibitor 2), M-CSF (macrophage colony-stimulating factor) and ICAM-1 (intercellular adhesion molecule-1). In cell culture of human oral epithelial cells and fibroblasts, it has been shown that hydrogel containing *Arenicola marina* hemoglobin (M101) does not show cytotoxic effects. Importantly, antibacterial activity towards *P. gingivalis* of this hydrogel has been demonstrated, making it a potential therapeutic option for optimization of non-invasive periodontitis treatment [150]. Molecule M101 displayed intrinsic Cu/Zn-SOD-like activity which was shown to be effective in reducing amanitin-induced hepatotoxicity when tested on progenitor HepaRG cells [151].

A commercially available product based on the extracellular hemoglobin of annelid *Nereis virens* aimed for use in cell culture as an oxygenating additive is HEMOXCell^®^. This product is described as non-immunogenic, can bind 156 oxygen molecules, and does not require a cofactor for oxygen delivery, allowing the release of oxygen in a simple gradient according to cell needs [74,76]. A cell culture oxygenating additive based on HEMOXCell^®^ increased the density of CHO-S cells (Chinese hamster ovary cells adapted to serum-free cultivation conditions) in a dose-dependent manner from 2.5 to 4.6 times in a concentration of 0.5 g/L, and also increased the production of recombinant proteins by these cells transfected with enhanced green fluorescent protein (EGFP) by 70%. This product also has SOD activity, which contributes to maintaining the quality of the cell culture medium [152]. The same authors showed that HEMOXCell^®^ at a concentration of 0.025 g/L increases the degree of proliferation of mesenchymal stem-stromal cells (MSCs) isolated from bone marrow by 25%, while preserving their clonogenic potential, phenotype, and plasticity [74]. Additionally, this product has been shown to increase MSC yield and stimulate osteogenic differentiation in a three-dimensional MSC culture in a bioreactor [153]. When compared with perfluorodecalin (artificial oxygen carrier), HEMOXCell^®^ performance is better in an in vitro model of rat islets, which makes it a preferable candidate in vivo application for islet encapsulation in a bioartificial pancreas [154].

## 6. Conclusions

Although the primary role of hemoglobin was discovered and explained more than a century ago, it is becoming increasingly recognized that extracellular hemoglobin has a prominent role in regulating several physiological conditions and the etiology and pathophysiology of numerous diseases (e.g., paroxysmal nocturnal hemoglobinuria sickle cell anemia, Alzheimer’s disease, multiple sclerosis, cerebral intraventricular hemorrhage, preeclampsia, etc.). The mechanism of action of endogenous extracellular hemoglobin in mammals is now well described. However, it is still necessary to take advantage of other emerging biological properties of this molecule and its derivatives to develop strategic approaches to control pathological processes. This is partially realized through applied biomedical research and the use of xenogeneic hemoglobin, such as bovine and porcine hemoglobin or hemoglobin of invertebrates, as cell culture additives and oxygen carriers. New discoveries in the field of extracellular hemoglobin research, which undertakes investigation on stem cells as well, should further clarify the capacity of extracellular hemoglobin to regulate cellular fates in vivo and may suggest, at the same time, a new approach to managing the application of hemoglobin molecules. Variation of hemoglobin concentration and optimal delivery of hemoglobin to maintain a suitable growing environment, by employing either encapsulation methods or more reliable 3D cultures such as multicellular organoids, is becoming an important element in the validity of future in vitro studies.
